# Intermediate-term trends in serum levels of metal ions after hip resurfacing arthroplasty

**DOI:** 10.1186/s13018-015-0335-0

**Published:** 2015-12-23

**Authors:** Wenxue Jiang, Yanlin Wan, Peng Cui, Xianjia Ning

**Affiliations:** Department of Orthopedics, Tianjin The First Center Hospital, 24 Fukang Rd., Nankai District, Tianjin, 300192 China; Department of Orthopedics, Tianjin Hospital, 406 Jiefang South Road, Hexi District, Tianjin, 300210 China; Department of Epidemiology, Tianjin Neurological Institute, 154 Anshan Rd, Heping District, Tianjin, 300052 China

**Keywords:** Hip resurfacing arthroplasty, Metal ion, Secular trends, Follow-up, Inductively coupled plasma mass spectrometry

## Abstract

**Background:**

The potential risks associated with hip resurfacing arthroplasty (HRA) are controversial and underestimated. The aim of this study was to explore intermediate-term trends for the levels of cobalt (Co), chromium (Cr), and molybdenum (Mo) ions after HRA.

**Methods:**

Forty patients who underwent HRA from October 2005 to December 2010 were recruited to this study. The serum levels of metal ions were examined preoperatively and 3, 12, 24, and 60 months after surgery. Trends and differences in levels of metal ions with respect to sex, operated side, and body mass index (BMI) were analyzed.

**Results:**

There were no significant differences in levels of Cr, Co, and Mo at each time point with respect to sex, operated side, and BMI (*p* > 0.05). The postoperative levels of Cr, Co, and Mo ions were significantly higher than the preoperative levels across sex, operated side, and BMI groups. Postoperative levels of Cr, Co, and Mo peaked at 12, 24, and 60 months, respectively. Cr levels peaked earlier (at 12 months) in the overweight (BMI ≥25 kg/m^2^) group compared to the normal-weight group (BMI <25 kg/m^2^), Co levels (at 12 months) peaked in women compared to men, and Mo levels (at 3 months) peaked in the bilateral HRA group compared to the unilateral HRA group.

**Conclusions:**

Serum levels of Cr, Co, and Mo increased significantly after HRA. Cr levels peaked earlier in the overweight patients, Co levels peaked in women, and Mo levels peaked in patients who underwent bilateral HRA. However, there were no significant differences with respect to sex, operated side, and BMI.

## Introduction

Hip resurfacing arthroplasty (HRA) has recently been extensively used in young, active patients with osteoarthritis or other degenerative diseases of the hip joint [1–5]. However, the metal-on-metal prosthesis gradually releases cobalt (Co), chromium (Cr), and molybdenum (Mo) ions. The metal ions may disseminate into the surrounding soft tissues and even into the body [6]. Currently, the factors influencing the serum levels of metal ions remain unclear.

Several adverse effects of metal ions have been reported, including local soft tissue reactions, pseudotumor formation, systemic effects, and elevated deposits of metal ions in target organs [7–11]. Studies performed in European and American countries showed that the location of the prosthesis is associated with the wear rate of the hip joint simulator [12]. However, the relationships between the levels of metal ions released following HRA and patient sex, operated side, and body mass index (BMI) remain unknown.

The purpose of the study was to assess the 5-year trends in the levels of metal ions released and to analyze the relationships between the levels of metal ions and patient sex, operated side, and BMI after HRA. This is the first report of 5-year changes in Cr, Co, and Mo levels in patients who had undergone HRA in China.

## Patients and methods

### Patient selection

The ethics committee of Tianjin First Central Hospital approved the study, and written informed consent was obtained from all participants during recruitment.

From October 2005 to December 2010, 40 patients who were diagnosed with osteoarthritis and inflammatory degenerative diseases of the hip joint underwent HRA performed by Wenxue Jiang in the Department of Orthopedics, Tianjin First Central Hospital, China. The diagnosis of osteoarthritis was established based on the patient’s symptoms and plain radiography performance; similarly, the diagnosis of inflammatory degenerative diseases was also based on the patient’s symptoms and plain radiography performance.

The inclusion criteria of the study were (1) patients with osteonecrosis of the femoral head with Association of Research Circulation Osseous (ARCO) grade IB (defined as the range of necrosis between 15 and 30 % on MRI), IC (defined as a range of necrosis of ≥30 % on magnetic resonance imaging (MRI)), IIC (defined as a range of necrosis of ≥30 % and the presence of a cystic lesion on MRI), and grade IV (defined as flattening of the femoral head, narrowing of the joint space, and sclerosis of the acetabulum on plain radiography; (2) patients with developmental dysplasia of the hip (DDH) with Crowe grade I (defined as dislocation of the femoral head of <50 %), Crowe grade II (defined as displacement of the proximal femur between 0.1 and 0.15 % of pelvic height), or Crowe grade III (defined as the displacement of proximal femur between 0.15 and 0.20 % of pelvis height or dislocation of femoral head between 75 and 100 %); (3) young, active patients between 20 and 64 years of age; (4) patients with severe hip joint pain that strongly affected the patient’s daily activities; (5) failed trials of nonoperative treatments; (6) patients with favorable bone mass based on the proximal femur; (7) patients with no deformities in the proximal femur; and (8) patients with normal liver and renal function [13].

The exclusion criteria were (1) severe proximal femur osteoporosis based on the Singh index (defined as Singh index ≥4) [14], (2) grade IV dysplasia based on the Crowe classification [15], (3) known allergy to metal, (4) bone tumors, (5) liver or renal dysfunction, and (6) patients with diabetes, gout, or other diseases. All assessments were performed by PC, who did not attend the treatments.

### Surgical procedure

**Fig. 1:**
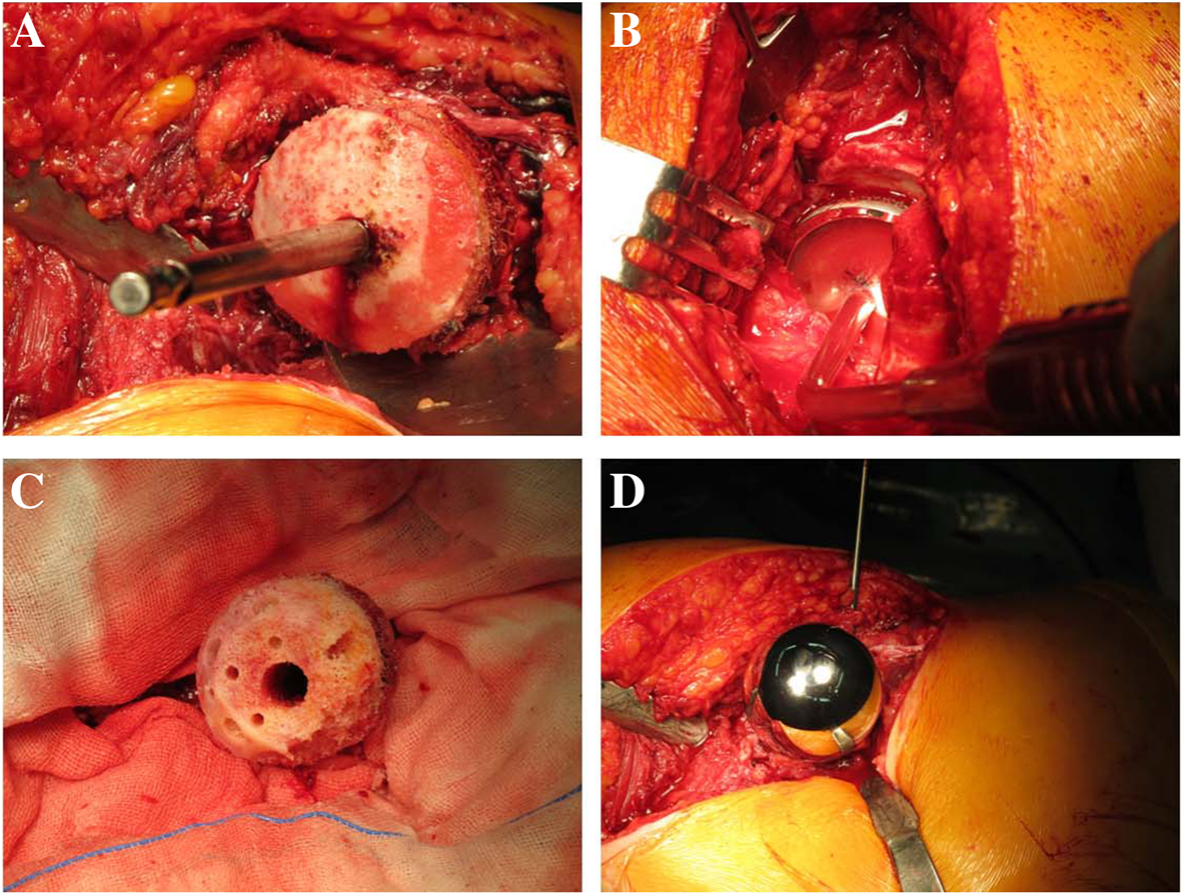
The process of surgical operation. **a** To perform borehole and bearizing on the center of the femur head under the conducting by a director. **b** To resect the acetabular labrum and burr until bleeding on the cartilage articularis, to impact the appropriate acetabular prosthesis with a position of 40° abduction and 20° anteversion, and to pressure for stabilization. **c** To burr the femur head using the barrel file and to resect the cystic lesion. **d** To embed the femoral prosthesis and fix with bone cement. Intraoperative photograph showing the embedded femoral prosthesis fixed with bone cement

**Fig. 2:**
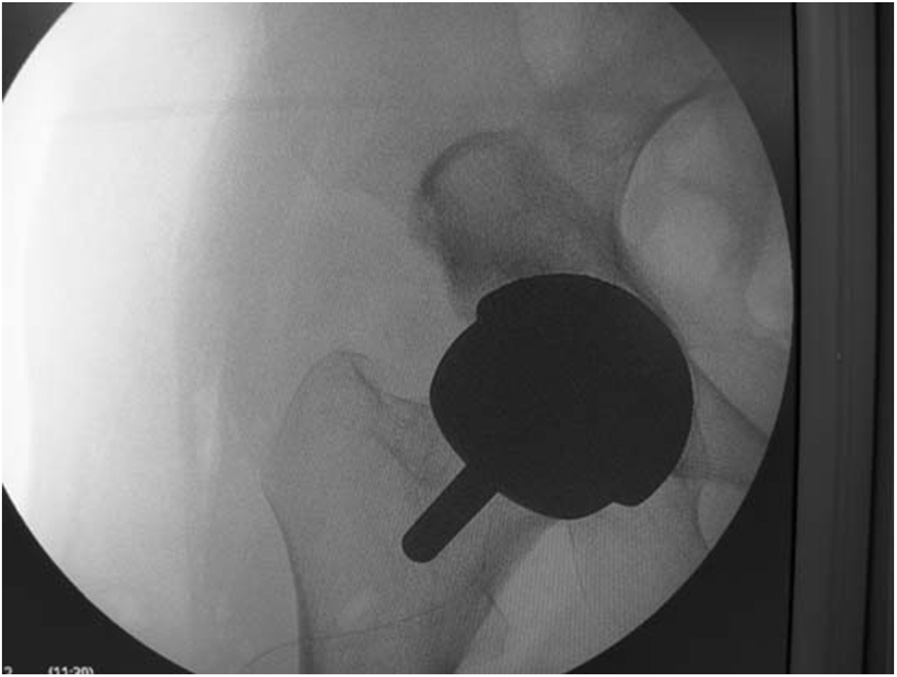
The X-ray plain film of prosthesis location. The X-ray plain film shows that the location of prosthesis is good during the operation. Postoperative radiograph showing typical hip resurfacing arthroplasty in the anteroposterior view

The operation was performed with the patient in the lateral decubitus position with the operated side facing up, as recommended by McMinn et al. [16]. After acetabular preparation, the acetabular component was impacted until fully seated, with a position of 40° abduction and 20° anteversion. Pressure was applied to stabilize the acetabular prosthesis. On the femoral head side, any cystic lesion was resected radically. The femoral prosthesis was inserted and fixed with bone cement. The femoral neck was supported with a band to avoid forming a notch at the junction of the femoral head and neck (Figs. 1 and 2).

### Measurement of metal ion levels in serum

Whole-blood samples were collected preoperatively and at 3, 12, 24, and 60 months postoperatively to assess the levels of Co, Cr, and Mo ions. The fasting blood sample was collected in a 10-ml plastic vacuum serum separator blood collection tube. The first 10 ml of blood was discarded to prevent metal contamination from the needle. The second 10 ml of blood was centrifuged at 4000 rpm for 5 min after incubating 30 min. The serum was drawn into a plastic tube with a sealed cap using a plastic suction device and was then stored at −20 °C. Subsequently, 0.5 ml of nitric acid was added to 0.5 ml of serum, and the mixture was incubated for 24 h; 4 ml of deionized distilled water was then added into the mixture and the volume was measured. After centrifuging at 8000 rpm for 3 min, the supernatant was removed and was used to measure the levels of Co, Cr, and Mo ions in triplicate. The serum levels of Co, Cr, and Mo ions were determined using an inductively coupled plasma mass spectrometer at the Experimental Center of Nankai University, China. We also assessed the preoperative renal function of all patients by measuring the serum urea and creatinine levels.

### Statistical analysis

Metal ion levels were expressed as the median and range, due to the asymmetrical distribution of the metal ion levels. Differences in the levels of metal ions between men and women, patients who had undergone unilateral and bilateral HRA, and those with body mass indexes (BMI) <25 kg/m^2^ and BMI ≥25 kg/m^2^ were analyzed using a nonparametric Mann-Whitney *U* test. Changes in time trends of metal ion levels were analyzed using the Kruskal-Wallis test. Age was represented as mean ± standard deviation. Statistical significance was set at *p* < 0.05. All statistical analyses were performed using SPSS software (version 15.0; SPSS Inc. Chicago, IL).

## Results

### Clinical characteristics of patients

In all, 40 patients were included in this study, including 19 (47.5 %) men and 21 (52.5 %) women. The mean age at surgery was 49.2 years overall (range, 26–64 years), 46.2 years in men (range, 26–58 years), and 52.0 years in women (range, 26–64 years), *p* = 0.062. The mean follow-up time was 24.1 months (range, 3–76 months). All patients underwent HRA with a metal-on-metal prosthesis. Twenty-eight patients (70 %) underwent bilateral resurfacing, and 12 (30 %) patients underwent unilateral resurfacing; there were no significant differences in the operated side between men and women (Table 1). The levels of serum urea and creatinine did not change during the study period.

**Table 1:** Demographic and clinical characteristics of patients

Characteristic	Men	Women	Total	*p*
Number, *n* (%)	19	21	40	–
Age, year, mean (SD)	46.2 (9.5)	52.0 (9.6)	49.2 (9.8)	0.062
BMI, kg/m^2^, mean (SD)	25.3 (3.7)	24.4 (2.6)	24.9 (3.2)	0.409
Diagnosis, *n* (%)				0.085
ONFH	9 (47.4)	6 (28.6)	15 (37.5)	
OA	2 (10.5)	0	2 (5)	
DDH	4 (21.1)	10 (47.6)	14 (35)	
RA	1 (5.3)	3 (14.3)	4 (10)	
AS	3 (15.8)	0	3 (7.5)	
Others	0	2 (9.5)	2 (4.0)	
Operated side, *n* (%)				0.836
Unilateral	13 (68.4)	15 (71.4)	28 (70)	
Bilateral	6 (31.6)	6 (28.6)	12 (30)	
Prosthesis company, *n* (%)				0.464
Conserve Plus	12 (63.2)	10 (47.6)	22 (55)	
BHR	2 (10.5)	6 (28.6)	8 (20)	
BMHR	3 (15.8)	4 (19)	7 (17.5)	
Depuy ASR-TM	2 (10.5)	1 (4.8)	3 (7.5)	

*SD* standard deviation, *BMI* body mass index, *ONFH* osteonecrosis of the femoral head, *OA* osteoarthritis, *DDH* developmental dysplasia of the hip, *RA* rheumatoid arthritis, *AS* ankylosing spondylitis

### Metal ion levels over time by sex, laterality, and BMI

As shown in Table 2, the levels of Cr, Co, and Mo at each time point were not significantly different when sex, operated side, and BMI groups were considered (*p* > 0.05). However, Cr levels were higher in women than in men (1.20 ng/ml vs. 1.88 ng/ml; *p* = 0.040) at 24 months postoperatively and in the BMI <25 kg/m^2^ group than in the BMI ≥25 kg/m^2^ group preoperatively (0.73 ng/ml vs. 0.66 ng/ml; *p* = 0.037).

**Table 2:** Levels of metal ions over time by sex, side, and body mass index

Time point	Cr	*p*	Co	*p*	Mo	*p*
Preoperation						
Men	0.70 (0.03)	0.949	0.72 (0.04)	0.949	0.82 (0.02)	0.564
Women	0.70 (0.03)		0.74 (0.03)		0.83 (0.02)	
Unilateral	0.68 (0.03)	0.478	0.72 (0.04)	0.848	0.82 (0.02)	0.564
Bilateral	0.72 (0.02)		0.74 (0.03)		0.83 (0.02)	
BMI <25 kg/m^2^	0.73 (0.02)	0.037	0.71 (0.02)	0.196	0.83 (0.01)	0.518
BMI ≥25 kg/m^2^	0.66 (0.02)		0.76 (0.04)		0.81 (0.02)	
3 months						
Men	1.06 (0.16)	0.668	1.55 (0.56)	0.116	6.26 (1.41)	0.253
Women	1.30 (0.29)		2.01 (0.57)		7.23 (0.88)	
Unilateral	1.15 (0.21)	0.237	1.97 (0.50)	0.866	6.16 (0.78)	0.176
Bilateral	1.31 (0.22)		1.21 (0.15)		8.86 (2.01)	
BMI <25 kg/m^2^	1.16 (0.22)	0.253	2.97 (1.05)	0.288	4.68 (0.56)	0.235
BMI ≥25 kg/m^2^	1.20 (0.22)		1.44 (0.36)		7.41 (0.92)	
12 months						
Men	1.80 (0.52)	0.487	2.16 (0.59)	0.298	6.91 (1.06)	0.817
Women	1.81 (0.4)		3.19 (0.72)		7.10 (0.78)	
Unilateral	1.67 (0.37)	0.713	2.32 (0.55)	0.327	7.35 (0.66)	0.540
Bilateral	2.06 (0.62)		3.50 (0.88)		6.33 (1.39)	
BMI <25 kg/m^2^	3.03 (1.47)	0.243	3.41 (1.86)	0.322	6.86 (3.65)	0.493
BM I ≥25 kg/m^2^	1.61 (0.28)		2.60 (0.50)		7.03 (0.6)	
24 months						
Men	1.20 (0.39)	0.04	2.96 (0.78)	0.661	4.67 (0.42)	0.242
Women	1.88 (0.30)		3.09 (0.59)		5.55 (0.86)	
Unilateral	1.53 (0.27)	0.236	3.00 (0.46)	0.693	4.84 (0.48)	0.324
Bilateral	2.08 (0.75)		3.26 (2.04)		7.23 (2.56)	
BMI <25 kg/m^2^	1.85 (0.45)	0.938	3.40 (0.74)	0.721	4.35 (0.74)	0.276
BMI ≥25 kg/m^2^	1.47 (0.3)		2.81 (0.60)		5.75 (0.74)	
60 months						
Men	1.17 (0.11)	0.584	3.60 (0.62)	0.361	7.42 (0.57)	0.584
Women	1.24 (0.28)		2.52 (0.720		7.60 (1.32)	
Unilateral	1.33 (0.28)	0.855	2.54 (0.70)	0.465	7.04 (1.25)	0.361
Bilateral	1.06 (0.06)		3.63 (0.66)		8.09 (0.67)	
BMI <25 kg/m^2^	1.29 (0.24)	1.000	2.80 (0.63)	0.850	7.12 (1.1)	0.345
BMI ≥25 kg/m^2^	1.06 (0.08)		3.45 (0.87)		8.22 (0.65)	

Data shown as mean (standard error)

### Trends for metal ion levels during the 5-year follow-up

Overall, the postoperative serum levels of Cr, Co, and Mo were significantly higher than the preoperative levels across sex, operated side, and BMI groups. Postoperative serum Cr levels peaked at 12 months, Co levels peaked at 24 months, and Mo levels peaked at 60 months. The respective ion levels peaked at 12, 60, and 60 months for men and 24, 12, and 60 months in women; 12, 24, and 12 months in the unilaterally operated group and 24, 60, and 3 months for the bilaterally operated group; and 24, 24, and 60 months for the BMI <25 kg/m^2^ group and 12, 60, and 60 months for the BMI ≥25 kg/m^2^ group (all *p* < 0.05). The trends for Cr, Co, and Mo levels before and after HRA are summarized in Table 3.

**Table 3:** Levels of metal ions over time by sex, laterality, and body mass index

Category	Preoperation	3 months	12 months	24 months	60 months
Total					
Cr, ng/ml	0.70 (0.02)	1.19 (0.17)^*^	1.80 (0.31)^*^	1.62 (0.25)^*^	1.20 (0.15)^*^
Co, ng/ml	0.73 (0.02)	1.79 (0.39)^*^	2.71 (0.48)^*^	3.04 (0.45)^*^	3.04 (0.49)^*^
Mo, ng/ml	0.82 (0.01)	6.78 (0.78)^*^	7.01 (0.62)^*^	5.21 (0.55)^*^	7.52 (0.73)^*^
Men					
Cr, ng/ml	0.70 (0.03)	1.06 (0.16)	1.8 (0.52)^*^	1.2 (0.39)^*^	1.17 (0.11)^*^
Co, ng/ml	0.72 (0.04)	1.55 (0.56)^*^	2.16 (0.59)^*^	2.96 (0.78)^*^	3.6 (0.62)^*^
Mo, g/ml	0.82 (0.02)	6.26 (1.41)^*^	6.91 (1.06)^*^	4.67 (0.42)^*^	7.42 (0.57)^*^
Women					
Cr, ng/ml	0.70 (0.03)	1.30 (0.29)^*^	1.81 (0.40)^*^	1.88 (0.30)^*^	1.24 (0.28)^*^
Co, ng/ml	0.74 (0.03)	2.01 (0.57)^*^	3.19 (0.72)^*^	3.09 (0.59)^*^	2.52 (0.72)^*^
Mo, g/ml	0.83 (0.02)	7.23 (0.88)^*^	7.1 (0.78)^*^	5.55 (0.86)^*^	7.6 (1.32)^*^
Unilateral					
Cr, ng/ml	0.68 (0.03)	1.15 (0.21)^*^	1.67 (0.37)^*^	1.53 (0.27)^*^	1.33 (0.28)^*^
Co, ng/ml	0.72 (0.04)	1.97 (0.5)^*^	2.32 (0.55)^*^	3.00 (0.46)^*^	2.54 (0.7)^*^
Mo, g/ml	0.82 (0.02)	6.16 (0.78)^*^	7.35 (0.66)^*^	4.84 (0.48)^*^	7.04 (1.25)^*^
Bilateral					
Cr, ng/ml	0.72 (0.02)	1.31 (0.22)	2.06 (0.62)^*^	2.08 (0.75)^*^	1.06 (0.06)^*^
Co, ng/ml	0.74 (0.03)	1.21 (0.15)	3.50 (0.88)^*^	3.26 (2.04)	3.63 (0.66)^*^
Mo, g/ml	0.83 (0.020	8.86 (2.01)^*^	6.33 (1.39)^*^	7.23 (2.56)	8.09 (0.67)^*^
BMI <25 kg/m^2^					
Cr, ng/ml	0.73 (0.02)	1.16 (0.22)	3.03 (1.47)	1.85 (0.45)^*^	1.29 (0.24)^*^
Co, ng/ml	0.71 (0.02)	2.97 (1.05)^*^	3.41 (1.86)	3.40 (0.74)^*^	2.8 (0.63)^*^
Mo, g/ml	0.83 (0.01)	4.68 (0.56)^*^	6.86 (3.65)	4.35 (0.74)^*^	7.12 (1.1)^*^
BMI ≥25 kg/m^2^					
Cr, ng/ml	0.66 (0.02)	1.20 (0.22)^*^	1.61 (0.28)^*^	1.47 (0.3)^*^	1.06 (0.08)^*^
Co, ng/ml	0.76 (0.04)	1.44 (0.36)^*^	2.60 (0.5)^*^	2.81 (0.6)^*^	3.45 (0.87)^*^
Mo, g/ml	0.81 (0.02)	7.41 (0.92)^*^	7.03 (0.6)^*^	5.75 (0.74)^*^	8.22 (0.65)^*^

Data shown as mean (standard error)

^*^Indicated *p* < 0.05 by nonparametric Kruskal-Wallis test. Data shown as mean (standard error)

### Complications and survival rate of prostheses after HRA

Only one patient experienced femoral neck fracture after HRA among all 40 patients during follow-up, which was pathologically diagnosed as osteonecrosis of the femoral head [10]. Except for this patient, no cases presented any complication, including an anaphylactic reaction to the metal ions, continuous pain in the groin, red/swollen skin and/or pruritus around the prosthesis, or malignancy. No surgery-related deaths occurred during the follow-up period, and the overall survival rate of the prostheses was 97.5 %.

## Discussion

In this study, we found that postoperative levels of Cr, Co, and Mo were significantly higher than preoperative levels across sex, operated side, and BMI groups. There were few significant differences in the levels of Cr, Co, and Mo at each time point by sex, operated side, and BMI groups, except that Cr levels were higher in women than in men 24 months postoperatively and in the BMI <25 kg/m^2^ group than in the BMI ≥25 kg/m^2^ group preoperatively. In addition, Cr levels peaked earlier in the BMI ≥25 kg/m^2^ group at 12 months, Co levels peaked in women at 12 months, and Mo levels peaked in the bilateral group at 3 months.

HRA has recently been extensively applied in young patients owing to a survival rate of 99 % and is the better option for young patients than is total hip replacement [17–24]. However, it has been established that the levels of metal ions in circulation in vivo increase persistently following HRA [25–27]. Researchers worldwide have noted this issue, though the long-term clinical effects remain undetermined.

Daniel et al. [28] analyzed the whole-blood levels of Co and Cr among 24 patients who had undergone unilateral HRA and found that Co and Cr levels were significantly increased after 1 year, followed by a decreasing trend until the sixth year. Yang et al. [25] assessed the levels of Co and Cr in 21 patients who underwent HRA and found a consistent increase in Co and Cr serum levels; Co and Cr levels peaked at 6 months (increased by 7.8-fold for Co and 10.1-fold for Cr) and at 9 months (increased by 4.7-fold for Co, and 7.8-fold for Cr), respectively, followed by a gradual decline after 6 months (levels were 3.6-fold for Co and 5.1-fold for Cr at 24 months). Levine et al. [17] found that the levels of Co and Cr ions were elevated in three implant groups (hybrid group, CoCr group, and titanium group) at all follow-up periods compared with those in the control group, and the mean Cr levels for the hybrid and CoCr groups were higher at 120 months compared to baseline levels, with increases of 3.9-fold and 3.6-fold, respectively. In contrast to these previous studies, we found that the postoperative levels of metal ions were elevated; the Cr level peaked at 12 months (increased by 1.6-fold) postoperatively, the Co level peaked at 60 months (increased by 3.2-fold), and the Mo level peaked at 60 months (increased by 8.2-fold). Compared to the results reported by Daniel et al. [27], our results demonstrated the same peak time for Cr levels and a delayed peak time for Co levels; however, the peak times of Cr and Co levels were delayed compared to those in the study reported by Yang et al. [26], in which the levels of Cr and Co peaked at 6 months postoperatively. The differences in peak time between the previous studies and our study may be explained by differences in prostheses [18], patients (young) [26], and types of operation (unilateral HRA) [28].

Previous studies showed that Cr levels were higher in women than in men [17, 18, 29], but we found no differences in levels of Co and Mo between men and women. In line with those studies, we observed an increased level of Cr in women. However, we did observe an earlier peak time for Co levels in women compared with men. This difference may be explained partly by the physiological differences between men and women.

The relationship of the levels of metal ions and the operated side for HRA is disputed. Moroni et al. [19] advocated that Cr levels were higher in patients who had undergone bilateral HRA compared with patients who had undergone unilateral HRA, though there was no difference in levels of Co and Mo. deSouza et al. [20] found that the level of Co was significantly increased (by 45 %) in patients who had undergone bilateral HRA. However, a few studies reported no changes in metal ion levels between those who underwent bilateral and unilateral HRA [21–23]. Consistent with the findings in these previous studies, there were no significant differences in metal ion levels between patients who had undergone bilateral and unilateral HRA in the present study. However, there was an earlier peak time for Mo ion level (3 months) in the bilateral HRA group compared to that in the unilateral HRA group. The reason is unknown.

Previous studies have demonstrated no significant relationship between BMI and metal ion levels after HRA [23, 30]. Consistent with these studies, we found no significant differences in metal ion levels between the normal-weight group and the overweight group postoperatively. However, Cr peaked earlier in the overweight group than in the normal-weight group. The increased load and wear of the hip joint in patients with a higher BMI after HRA may partly explain the earlier peak for Cr levels in the overweight group than in the normal-weight group [23].

In this study, metal ion levels were measured using inductively coupled plasma mass spectrometry that is associated with high accuracy and less interference and has been extensively applied to measure metal ion levels. There are several limitations in this study. First, there was a small sample size, as only 40 patients were assessed. Second, over the follow-up period of 5 years, measurements were only performed four times. Therefore, further studies with a greater number of postoperative measurements and with larger numbers of patients are necessary to confirm these findings.

## Conclusion

Our study suggests that serum levels of Cr, Co, and Mo increase significantly after HRA. Cr levels peaked earlier in the overweight patients, Co levels peaked in women, and Mo levels peaked in patients who underwent bilateral HRA. However, there were no significant differences with respect to sex, operated side, and BMI. A long-term study on the levels of metal ions is necessary to improve understanding of the effects of metal ions after HRA.
